# Depression, Insomnia, and Probable Post-Traumatic Stress Disorder among Survivors of the 2016 Kumamoto Earthquake and Related Factors during the Recovery Period amidst the COVID-19 Pandemic

**DOI:** 10.3390/ijerph19074403

**Published:** 2022-04-06

**Authors:** Ayako Ide-Okochi, Tomonori Samiso, Yumie Kanamori, Mu He, Mika Sakaguchi, Kazumi Fujimura

**Affiliations:** 1 Graduate School of Health Sciences, Kumamoto University, Kumamoto City 862-0976, Japan; yumiek@kumamoto-u.ac.jp (Y.K.); 205w9101@st.kumamoto-u.ac.jp (M.H.); 217w0102@st.kumamoto-u.ac.jp (M.S.); 2 Health and Welfare Policy Division, Health and Welfare Bureau, Kumamoto City 860-0808, Japan; samiso.tomonori@city.kumamoto.lg.jp; 3 Department of Community Health Systems Nursing, Ehime University Graduate School of Medicine, Toon City 791-0295, Japan; fujimura.kazumi.ox@ehime-u.ac.jp

**Keywords:** earthquake, depression, insomnia, post-traumatic stress disorder, loneliness, housing, social relationships, COVID-19, recovery

## Abstract

The aftereffects of the severe 2016 Kumamoto earthquake were complicated by the COVID-19 pandemic. This study aimed to identify mental health problems and related factors among survivors five years after the earthquake and clarify its long-term effects. A cross-sectional survey was conducted in 2020 among 19,212 survivors affected by the earthquake who moved from temporary to permanent housing. We analysed 8966 respondents (5135 women, 3831 men; mean age 62.25 ± 17.29 years). Logistic regression analysis was conducted to examine associations between mental health problems and socioeconomic factors. Prevalence rates of psychological distress, insomnia, and probable post-traumatic stress disorder were 11.9%, 35.2%, and 4.1%, respectively. Female gender (OR = 1.33, 95% CI = 1.13–1.57; OR = 1.21, 95% CI = 1.08–1.34; OR = 1.81, 95% CI = 1.41–2.32), public housing (OR = 2.14, 95% CI = 1.63–2.83; OR = 1.54, 95% CI = 1.26–1.88; OR = 2.41, 95% CI = 1.62–3.58), loneliness (OR = 9.08, 95% CI = 7.71–10.70; OR = 5.55, 95% CI = 4.90–6.30; OR = 3.52, 95% CI = 2.77–4.49), COVID-19-induced activity reduction (OR = 1.41, 95% CI = 1.19–1.66; OR = 1.86, 95% CI = 1.68–2.07; OR = 1.80, 95% CI = 1.40–2.31), and COVID-19-induced income reduction (OR = 1.33, 95% CI = 1.12–1.57; OR = 1.43, 95% CI = 1.28–1.59; OR = 1.92, 95% CI = 1.51–2.43) were significantly associated with mental health problems. These results suggest that gender, current housing, loneliness, and COVID-19 affected the survivors’ mental health during recovery.

## 1. Introduction

A sequence of two strike-slip earthquakes occurred on 14 and 16 April 2016 in the intraplate region of Kyushu Island, Japan, and caused significant damage and disruption to the Kumamoto region. The Kumamoto earthquake caused seven magnitude 5.4–7.3 earthquakes over three days from 14–16 April in 2016 [1]. It was the first time that a seismic intensity of 7 (on the Japan Meteorological Agency scale) was recorded twice within two days in Japan [2,3]. In Kumamoto Prefecture, aftershocks felt by the human body continue [4], with more than 4364 tremors in the 15 months since the earthquake [2]. This earthquake characteristic affected the health of survivors. The number of certified disaster-related deaths from indirect causes, such as venous thrombosis, post-traumatic stress disorder (PTSD), stress during the evacuation phase, and suicide was four times the number of direct deaths caused by the earthquakes [5].

In Kumamoto City, the largest city in Kumamoto Prefecture, up to 110,000 people of an approximate population of 700,000 people were evacuated [6]. In April 2021, five years had passed since the Kumamoto earthquake struck. By 2020, survivors living in temporary housing in Kumamoto City have primarily moved to permanent housing. However, the long-term mental health impacts on earthquake survivors remain a concern [7]. Studies have shown that post-traumatic stress symptoms gradually decreased over time in areas affected by the Great East Japan Earthquake (GEJE) [7,8]. Note that GEJE is a composite of natural and nuclear disasters [6], and this study refers to previous studies mainly conducted in Miyagi Prefecture targeting victims of natural disasters. However, previous studies have reported no such tendency for other psychiatric disorders [7]. In addition, a survey of Kumamoto earthquake survivors conducted in 2016 found that a decrease in social capital after the Kumamoto earthquake significantly increased the risk for depression (major depressive episode) in women [9]. Therefore, we were concerned about the long-term effects of the disaster on the mental health of Kumamoto earthquake survivors. However, few studies have revealed the mental health status of Kumamoto earthquake survivors during the recovery period. Eight years after the 2011 Van earthquake in Turkey, 8.9% of survivors had prolonged grief disorder. Female gender, age 40 years or older, personal injury, property damage, loss of family members, and the psychological impact of the earthquake were associated with the occurrence of prolonged grief disorder [10]. It is possible that mental health problems also occurred in survivors of the Kumamoto earthquake during the recovery period. However, there have been only a few studies on sleep problems among survivors after aftershocks lasting more than six months, with the exception of residents of a Greek island [11]. Therefore, their current situation should be analysed and mental health-related factors identified.

Furthermore, these survivors experienced additional adverse events during the recovery period of the Kumamoto earthquake. The final stages of their migration from temporary housing occurred during a pandemic as the World Health Organization (WHO) declared on 11 March 2020 coronavirus disease 2019 (COVID-19) a pandemic [12]. In Japan, a nationwide state of emergency was declared on 16 April 2020, urging people to refrain from leaving their homes. Such behavioural restrictions affect health, safety, and well-being and can cause emotional distress and unhealthy behaviours [13]. Displaced people with mental disorders showed worsening symptoms even before the COVID-19 epidemic [14]. At the same time, the general population reported worsening psychological well-being and increased anxiety and depression [14]. Survivors suffering from the psychological burden of natural disasters are expected to experience an even greater psychological burden due to the social and economic changes of the COVID-19 pandemic [15,16]. Thus, new factors imposed by the pandemic during the recovery period after the earthquake may have had a profound impact on earthquake survivors who had mental health problems or were still recovering from the sequalae of the Kumamoto earthquake. Reports amid double disasters (COVID-19 and natural disasters) from Croatia [17], the Philippines [16], and Japan [15] described the concerns of the negative impact on mental health and preventive measures. However, few studies have empirically examined the relationship between the pandemic and the mental health status using scales among earthquake survivors during their recovery period. In addition, there is little research on factors related to the mental health of disaster survivors in double disasters.

Regarding the long-term mental health of earthquake survivors, PTSD, depression, anxiety, problematic behaviours, and sleep problems all have generally high prevalence rates and are persistent in this population [7]. A study found bidirectional relationships between insomnia, PTSD, and depressive symptoms among 1492 adolescent survivors of the 2008 Wenchuan earthquake in China [18]. Most risk factors for mental health problems after the 2011 GEJE were related to the reorganisation of daily lives and social networks [7]. However, previous studies have not thoroughly examined the factors associated with mental health problems among survivors of the Kumamoto earthquake after they migrated from temporary housing. Restoration of their lives was probably difficult due to the prolonged period with aftershocks. Therefore, this study aimed to analyse mental health problems among earthquake survivors and identify related factors, including changes caused by COVID-19, during their recovery period. We hypothesized that the prevalence of depression, insomnia, and probable PTSD symptoms would be high among Kumamoto earthquake survivors, and that these mental health problems would be associated with social relationship status, housing conditions, and life changes due to COVID-19.

## 2. Materials and Methods

### 2.1. Participants

All adult survivors (19,212 persons) of the 2016 Kumamoto earthquake participated in this study, who met the following conditions: must move out of temporary housing by December 2019 and reside in Kumamoto City in 2020. From the city’s earthquake survivors’ registry, we extracted the addresses of household heads and the number of adult family members over 18 years of age. The total population of Kumamoto City in 2020 was 738,567 comprising 348,684 men and 389,883 women [19]. In 2020, the population aged 18–64 years was 420,090 (68.1%), and the population aged 65 years and over was 196,435 (31.9%) [19].

This was a cross-sectional study that used a self-administered questionnaire. The questionnaire contained 49 items. We mailed 11,479 households questionnaires, instructions and returned envelopes for the number of subjects selected in that household. In an instruction sheet, we described the reasons for selection, assurance of free-will participation, no disadvantage in the case of refusal, protection of personal information, the possibility of discomfort from remembering the disaster, publication of results, and contact information in case of any help for answering the questionnaire. Moreover, we explained a returned questionnaire was regarded as consent. Individuals in each family completed the questionnaires and returned them individually by mail. The data collection period was from July to December 2020. The questionnaire return rate was 49.0%. We excluded 130 questionnaires returned without answers and 313 questionnaires with more than 95% of the questions left unanswered. We analysed data from 8966 persons. The return of the written questionnaire was considered as consent [20]. 

### 2.2. Mental Health Problems

#### 2.2.1. Psychological Distress (K6)

This study assessed psychological distress using the Kessler psychological distress scale (K6). K6 is a scale developed by Kessler et al. to screen for mental disorders, such as depression and anxiety [21]. The Japanese version of K6 has been validated previously [22]. It consists of six questions about how often an individual has felt the following in the last month: (1) nervous, (2) hopeless, (3) restless or fidgety, (4) so sad that nothing could cheer them up, (5) everything was an effort, and (6) worthless. The total K6 score ranges from 0 to 24. Previous studies used the three cutoff points 5, 10, and 13 to screen for psychological stress [23]. Similar to the Comprehensive Survey of Living Conditions conducted by the Ministry of Health, Labour and Welfare [24] and other studies regarding mental health problems after the GEJE [8,25,26,27], we classified participants with K6 scores ≥10 as having high psychological distress equivalent to mood disorder or anxiety disorder.

#### 2.2.2. Insomnia (Athens Insomnia Scale)

Sleeping disorders were assessed using the Japanese version of the Athens Insomnia Scale (AIS-J) [28]. Whereas the original AIS is a self-rating inventory consisting of eight items [29], the AIS-J consists of a two-factor structure: “nocturnal sleep problem” and “daytime dysfunction”. The AIS-J is rated on a four-point scale (0 = no problem at all, 1 = slightly problematic, 2 = markedly problematic, and 3 = extremely problematic). The AIS-J scores range from 0 to 24, with a score of 6 or more indicating insomnia [30]. Participants were required to place their subjective judgment of symptom positivity (1, 2, and 3) if they had experienced sleep difficulties at least three times per week during the preceding month.

#### 2.2.3. Probable PTSD

We assessed self-reported PTSD symptoms using the Japanese version of the Posttraumatic Diagnostic Scale (PDS) [31], which was developed for a brief screening of PTSD [32]. PTSD3 is a short version of the Japanese version of the PDS [33]. The Japanese versions of both PDS and PTSD3 have been validated. Previous surveys of GEJE survivors and Kumamoto Earthquake survivors have been conducted over time using the questions based on PTSD3 since 2011 [34,35,36,37,38]. Traumatic events (“intrusive images”, “nightmares”, and “physiological reactions when reminded of the trauma”) were reported at least twice in the past week. We considered participants who answered “yes” to two or more of the three items on the PTSD3 to be at risk for probable PTSD according to the criteria adopted by Kumamoto Prefecture [36].

### 2.3. Other Variables

#### 2.3.1. Attributes

We analysed the following attributes: gender, age, cohabitants, mental illness, and treatment status. The mental illness here is separate from the aforementioned psychological distress, insomnia, and probable PTSD, and we asked the respondents to self-report the presence or absence of illness. After calculating the average age and range, we divided the participants into two groups: ≤64 years and ≥65 years.

#### 2.3.2. Housing Conditions

Participants were asked about their temporary housing category, their current residence after leaving temporary housing (permanent housing), and whether they had changed their elementary school district due to resettlement. The temporary housing categories were prefabricated temporary housing, temporary housing in the private/public rented sectors. After the Kumamoto earthquake, the municipalities operated private and public rented accommodations as temporary housing. As permanent housing for those who lost their homes in the Kumamoto earthquake and found it difficult to rebuild or rent an accommodation on their own, post-disaster public housing was built by the Kumamoto City government. Compared to other public housing, disaster public housing is considered to have a higher percentage of disaster victims. Currently, people live in their own homes, rented housing, public housing, post-disaster public housing, hospitals, retirement homes, and others. A change in school district means relocation, a risk factor for the mental health problems [39]. Survivors of who relocated after the 2011 Christchurch earthquake were significantly more likely to seek treatment for mood and anxiety symptoms than those who remained at their pre-disaster address [39]. In Kumamoto City, the school district is the basic unit for health promotion and community building [40].

#### 2.3.3. Social Relationships

We asked the study participants to indicate their participation in events and social gatherings held in the community by indicating “I participate”, “I do not participate”, or “I do not know about such events”. Next, they were asked to rate on a four-point scale (never, hardly ever, sometimes, always) if they ever felt isolated from others. For the analysis, “never” and “hardly ever” were categorised as the absence of loneliness, whereas “sometimes” and “always” were classified as its presence.

#### 2.3.4. Self-Reported Impact of COVID-19

We asked participants to indicate on a three-point scale (increase, no change, or decrease) the change in their current opportunities for social and physical activity compared with those before the COVID-19 outbreak. An “increase” or “no change” was categorized as no decrease in opportunities. Participants were also asked whether their income had decreased due to the COVID-19 outbreak. For the analysis, “decreased significantly” and “decreased slightly” were categorised as indicating a decrease in income, whereas “did not decrease” indicated no decrease in income.

### 2.4. Data Analysis

Descriptive statistics were calculated for all variables. First, the chi-square test of independence (χ^2^ test) was performed to examine the association between the presence of mental health problems and the variables of attributes, housing conditions, social relationships, and the impact of COVID-19. Second, a binary logistic regression analysis was performed to examine the factors influencing mental health problems. The independent variables were gender, age, presence of co-residents, temporary housing category, current residence (permanent housing), school district change, community participation, loneliness, a decrease in socialising due to COVID-19, and a decrease in income due to COVID-19. Odds ratios (ORs) and 95% confidence intervals (95% CIs) were used to examine the results. Before logistic regression analysis, Spearman’s rank correlation coefficients were calculated to check for multicollinearity among the independent variables and ensure that the correlation coefficients did not indicate a strong correlation (|r| > 0.4). Variable selection was performed using the forced imputation method. We assessed the model’s goodness of fit using the Hosmer-Lemeshow test. For each variable, missing values were excluded from the analysis. Statistical analysis was performed using IBM SPSS 28 for Windows (IBM Corp., Armonk, NY, USA) with a significance level of *p* < 0.05.

## 3. Results

Table 1 shows the demographic characteristics of the participants comprising 5135 (57.3%) women and 3831 (42.7%) men. The mean age of the study population was 62.25 ± 17.29 years (18–105 years), and 53.1% of the participants were aged 65 years or above. In Japan, people aged 65 years or older are considered elderly. Among the study participants, 78.9% lived with another person, 56.6% lived in their own homes. A total of 34.2% were forced to leave their pre-earthquake school districts when they moved into permanent housing. A total of 22.2% of the participants reported community participation. Of the study participants, 20.8% felt lonely. Table 2 shows the prevalence rates of mental health problems. Psychological distress, insomnia, and probable PTSD were found in 11.9%, 35.2%, and 4.1% of the participants, respectively.

Table 3 shows the results of the χ^2^ tests between independent variables and the presence or absence of each mental health problem. Table 4 shows the results of the binary logistic regression analyses with the presence of mental health problems as the dependent variable and attributes, social relationships, and COVID-19 effects as independent variables. In all logistic regression analyses, the results of the χ^2^ test for the model were significant at *p* < 0.05, and the results of the Hosmer-Lemeshow test were *p* ≥ 0.05.

Participants with female gender (OR = 1.33, 95% CI = 1.13–1.57), rented housing (OR = 1.50, 95% CI = 1.21–1.87), public housing (OR = 2.14, 95% CI = 1.63–2.83), public housing for disaster survivors (OR = 2.16, 95% CI = 1.08–4.32), loneliness (OR = 9.08, 95% CI = 7.71–10.70), lack of community participation (OR = 1.74, 95% CI = 1.3–26.22), not knowing event information (OR = 1.84, 95% CI = 1.36–2.50), reduced activity due to COVID-19 (OR = 1.41, 95% CI = 1.19–1.66), and reduced income due to COVID-19 (OR = 1.33, 95% CI = 1.12–1.57) were more likely to experience psychological distress. Insomnia was more likely to be associated with female gender (OR = 1.21, 95% CI = 1.08–1.34), age of 65 years or above (OR = 1.36, 95% CI = 1.22–1.52), living alone (OR = 1.20, 95% CI = 1.04–1.38), public housing (OR = 1.54, 95% CI = 1.26–1.88), loneliness (OR = 5.55, 95% CI = 4.90–6.30), lack of community participation (OR = 1.53, 95% CI = 1.34–1.75), not knowing event information (OR = 1.56, 95% CI = 1.29–1.89), decreased activities due to COVID-19 (OR = 1.86, 95% CI = 1.68–2.07), and decreased income due to COVID-19 (OR = 1.43, 95% CI = 1.28–1.59). Probable PTSD was more likely to be associated with female gender (OR = 1.81, 95% CI = 1.41–2.32), age of 65 years or above (OR = 1.49, 95% CI = 1.16–1.90), rented housing (OR = 1.95, 95% CI = 1.41–2.69), public housing (OR = 2.41, 95% CI = 1.62–3.58), post-disaster public housing (OR = 2.75, 95% CI = 1.23–6.16), no change in school district (OR = 2.07, 95% CI = 1.01–4.28), loneliness (OR = 3.52, 95% CI = 2.77–4.49), reduced activities caused by COVID-19 (OR = 1.80, 95% CI = 1.40–2.31), and reduced income caused by COVID-19 (OR = 1.92, 95% CI = 1.51–2.43).

## 4. Discussion

To the best of our knowledge, this is the first study to evaluate long-term mental health problems and identify related factors, including COVID-19-related changes, five years after large earthquakes with magnitudes exceeding 7. The results of this study provide a guideline for the long-term prognosis and preventive measures for the mental health of earthquake survivors.

### 4.1. Prevalence Rates of Mental Health Problems

The prevalence of a K6 score ≥ 10 was 9.6% among 3743 public servants in the Miyagi Prefectural Government seven months after the GEJE [26]. Two years after the GEJE, the proportion of survivors with psychological distress (K6 score ≥ 10) in one city in Miyagi Prefecture ranged from 0–20% [27]. In a survey conducted in two cities five years after the GEJE, the percentages of respondents with K6 scores ≥ 10 were 12.6% and 17.3%, respectively. Furthermore, in a study conducted in 2020 in Kumamoto Prefecture, this percentage was 13.9% among Kumamoto earthquake survivors [36]. Thus, similar to victims of the GEJE and Kumamoto earthquake, the psychological distress rate was high among our study participants even five years after the earthquake.

The percentages of participants with insomnia (AIS-J score ≥ 6) were 31.4% and 38.0% in a survey of disaster survivors in two areas of Miyagi Prefecture conducted five years after the GEJE [35]. Among Japanese blue- and white-collar men, 18.8% and 18.3%, respectively, had a score ≥ 6 on the Athens Insomnia Scale [30]. Thus, the proportion of participants with insomnia was in our study as high as that of survivors of the GEJE who were additionally hit by a 15-m-high tsunami and was significantly higher than that of male workers under normal conditions. Earthquakes are traumatic events that cause acute and lasting stress to survivors [11]. The Kumamoto earthquake had frequent aftershocks [1,3] and Kumamoto City was very close to the epicentre at 4.4 km [41]. The relatively high insomnia prevalence in our study might have been caused by the continuing worry due to frequent aftershocks [11] and the proximity to epicentre [42].

According to a review of mental health problems in survivors of the GEJE, a survey conducted nine months after the earthquake [43] showed the longest-lasting effects [7]. In the above study, 1180 high school students were surveyed using the Japanese version of the Impact of Event Scale-Revised (IES-R), and the proportion of students with an IES-R score ≥ 25 was 10% [43]. Eight years after the Wenchuan earthquake in China, 11.8% of the adult participants had PTSD symptoms as assessed by the IES-R [44]. A review of mental health problems among survivors of the GEJE found a trend toward decreased PTSD symptoms over time [7]. Since the present study was conducted five years after the earthquake, the prevalence may have decreased, although the comparability of our probable PTSD data with those of other studies was limited since our assessment tool differs from that of the cited studies.

### 4.2. Factors Associated with Mental Health Problems

Female gender, current residence in public housing, loneliness, and reduced activity and income due to COVID-19 were all associated with psychological distress, sleep disturbance, and PTSD risk. In this study, we limit our discussion to these factors.

#### 4.2.1. Attributes

Female gender was significantly associated with mental health problems. In a review article on mental health problems after the GEJE, being a woman was identified as a risk factor for PTSD and depression [7]. In a survey conducted annually from 2011 to 2018 among GEJE survivors, women were consistently more likely than men to have psychological distress, sleep disturbance, and PTSD risk [35]. Furthermore, being female has been associated with depression and anxiety during the COVID-19 pandemic [45]. The findings of our study are consistent with those of previous studies and suggest that it is necessary to consider gender differences in mental health measures for earthquake survivors.

#### 4.2.2. Type of Permanent Housing

Being in public housing as the current residence was significantly associated with all mental health problems assessed in the present study. Existing research has examined the association between housing type and mental health among survivors of the GEJE. Two and a half years after the GEJE, the adjusted rate ratio for depressive symptoms among older survivors was about twice as high among residents of prefabricated temporary housing as those living in other housing types [46]. However, the association between the type of permanent housing and mental health after leaving temporary housing has not been fully investigated before. The present study is one of the few studies to clarify the association between housing type and mental health during the recovery period after moving out of temporary housing. Due to the continuing mental health problems in earthquake survivors [7], long-term studies are needed to examine the association between permanent housing type and mental health after leaving temporary housing.

#### 4.2.3. Social Relationships

Loneliness was associated with a higher risk for psychological distress, sleep disturbances, and probable PTSD. The odds ratio for psychological distress was exceptionally high, indicating a robust association. Social networks and social capital have been investigated with regard to health and social relationships among earthquake survivors. Two years after the GEJE, social isolation measured using the Lubben Social Network Scale 6 was significantly associated with depressive symptoms evaluated using the Center for Epidemiological Studies-Depressive Scale [47]. Among survivors of the GEJE, those who considered community trust weak in 2011 had significantly higher Athens Insomnia Scale scores, a measure of sleep disturbance, for the entire period up to 2017 [48]. However, little is known about its association between insomnia and loneliness. People with a high sense of loneliness have less social contact and spend more time alone [49], which is associated with social isolation. Therefore, loneliness poses a severe health risk. The present study is one of the few to reveal the relationship between loneliness and mental health problems among survivors five years after an earthquake. Loneliness is thought to have a medium-to-long-term impact on the health of survivors. It is essential to conduct long-term research on loneliness, isolation, social capital, and mental health issues to provide better support. Research on solitary deaths among disaster victims in Japan suggests preventing their isolation by enabling them to conduct daily living activities such as eating and bathing in a communal setting [50]. It is important that sharing life in small community units based on school districts is promoted on the initiative of residents [16].

#### 4.2.4. Factors Associated with the COVID-19 Pandemic

According to our study results, COVID-19-induced decreases in activity and income were significantly associated with mental health problems. A review of the quarantine effects in relation to COVID-19 found that people and healthcare workers who were isolated due to infectious diseases such as SARS (severe acute respiratory syndrome), H1N1 influenza, and Ebola showed emotional responses such as stress, depression, irritability, insomnia, fear, confusion, anger, frustration, boredom, and feelings of stigmatisation [51]. Stressors included the duration of isolation and self-restraint, inadequate supplies of necessities, difficulties in securing medical care and medicines, and economic losses due to isolation and self-restraint [51]. In the present study, the reduction in activity and income due to COVID-19 may have acted as a stressor and contributed to exacerbating mental health risks among earthquake survivors [15]. In practice for GEJE survivors, telephone counselling was provided for preventing mental health deterioration due to the restrictions on social and economic activities and anxiety caused by COVID-19 added to the severe conditions surrounding the survivors [15]. Although research on the health effects of infectious disease pandemics among earthquake survivors is lacking, the occurrence of the COVID-19 pandemics and the resulting contact restrictions can be seen as an additional burden following the Kumamoto earthquake. To prevent long-term damage to the health of earthquake survivors, we need to be aware of the new stressor imposed by the COVID-19 pandemics and provide support to survivors.

### 4.3. Limitations and Significance of the Study

First, this study was limited to Kumamoto earthquake survivors, and the results obtained may not apply to all survivors of earthquakes. Second, as it is a cross-sectional study, it is impossible to prove causality. In particular, there is little empirical research on the effects of COVID-19 on earthquake survivors during the recovery period, although various interactions are conceivable. Longitudinal studies are needed to explain the causal relationships. Third, the questions regarding probable PTSD symptoms are a simple assessment of risk, not a definitive diagnosis. In the future, a comprehensive PTSD scale that reflects the Diagnostic and Statistical Manual of Mental Disorders (DSM) criteria [14,18] should be used to more accurately measure the risk of PTSD in survivors. Fourth, other vital determinants besides the variables included in the model are possible. In future studies, it is necessary to increase the number of socioeconomic items and adopt a multidimensional view. Fifth, future surveys of disaster survivors should use methods that more clearly confirm informed consent. Despite these limitations, it is of importance that this study clarified the mental health problems and related factors after survivors had moved from temporary housing to permanent housing five years after the Kumamoto earthquake, thereby providing knowledge for planning future support.

## 5. Conclusions

Five years have passed since the Kumamoto earthquake, but the mental health indicators of survivors were still unusually low, showing the continuing effects of the earthquake. Among depression, insomnia, and probable PTSD, the prevalence of insomnia was extremely high, possibly due to the impact of continued aftershocks. The odds ratio for loneliness was the highest among factors associated with mental health problems. In addition, COVID-19-induced life changes were significantly associated with mental health problems. During recovery from the earthquake effects, mental health risks were still increased, and the COVID-19 pandemic has caused an additional burden. Research and long-term support are essential to prevent adverse outcomes in Kumamoto earthquake survivors.

## Figures and Tables

**Table 1:** Demographics of the participants.

			*n* = 8966
		*n*	%
Gender	Male	3831	42.7
	Female	5135	57.3
Age	Mean ± sd (years)	62.25	±17.29
	18–64 years old	4208	46.9
	65 years old	4758	53.1
Cohabitant	None	1805	20.1
	Yes	7077	78.9
Temporary housing category	Prefabricated temporary housing	544	6.1
	Temporary housing in the private sectors	7892	88.0
	Temporary housing in the public sectors	528	5.9
Current residence	Owned house	5072	56.6
	Houses for rent	2383	26.6
	Public housing	1116	12.4
	Public housing for disaster	116	1.3
	Hospitals and institutions	66	0.7
	Other	159	1.8
Change of residential school district	None	5462	61.8
	Yes	3065	34.7
	I don’t know	312	3.5
Mental illness	None	5687	63.4
	Yes	469	5.2
Loneliness	None	6913	77.1
	Yes	1867	20.8
Community participation	No information of such events	1111	12.4
	None	5677	63.3
	Yes	1991	22.2
Decrease in activity opportunities due to COVID-19	None	4418	49.3
	Yes	4316	48.1
Decrease in income due to COVID-19	None	5533	61.7
	Yes	2956	33.0

COVID-19: coronavirus disease 2019.

**Table 2:** Prevalence rates of mental health problems among study participants.

			*n* = 8966
		*n*	%
K6 score			
	K6 ≥ 5	2663	32.8
	K6 ≥ 10	964	11.9
	K6 ≥ 13	428	5.3
Psychological distress (K6 ≥ 10)			
	None	7158	88.1
	Yes	964	11.9
Insomnia (AIS-J ≥ 6)			
	None	5398	64.8
	Yes	2932	35.2
Probable PTSD			
	None	8601	95.9
	Yes	365	4.1

K6: the Kessler psychological distress scale; AIS-J: the Japanese version of the Athens Insomnia Scale; PTSD: post-traumatic stress disorder.

**Table 3:** Cross-tabulation of mental health problems and independent variables.

																*n* = 8966
		Psychological Distress					Insomnia					Probable PTSD				
		Applicable		Not Applicable			Applicable		Not Applicable			Applicable		Not Applicable		
		*n* = 964		*n* = 7158		*p* Value	*n* = 2932		*n* = 5398		*p* Value	*n* = 365		*n* = 8601		*p* Value
		*n*	%	*n*	%		*n*	%	*n*	%		*n*	%	*n*	%	
Gender						<0.001					<0.001					<0.001
	Male	354	36.7	3140	43.9		1168	39.8	2436	45.1		111	30.4	3720	43.3	
	Female	610	63.3	4018	56.1		1764	60.2	2962	54.9		254	69.6	4881	56.7	
The elderly						0.304					<0.001					0.002
	None (18–64 years old)	499	51.8	3579	50.0		1326	45.2	2735	50.7		142	38.9	4066	47.3	
	Yes (over 65 years)	465	48.2	3579	50.0		1606	54.8	2663	49.3		223	61.1	4535	52.7	
Live-in						<0.001					<0.001					<0.001
	Yes	667	69.6	5843	82.0		2172	74.6	4492	83.6		252	69.4	6825	80.1	
	None	292	30.4	1282	18.0		739	25.4	881	16.4		111	30.6	1694	19.9	
Temporary housing category						<0.001					<0.001					0.016
	Prefabricated temporary housing	58	6.0	410	5.7		171	5.8	318	5.9		30	8.2	514	6.0	
	Temporary housing (rental)	810	84.1	6385	89.2		2536	86.5	4824	89.4		304	83.3	7588	88.2	
	Temporar housing (public sectors)	95	9.9	362	5.1		224	7.6	255	4.7		31	8.5	497	5.8	
Current residence						<0.001					<0.001					<0.001
	Owned house	382	39.7	4295	60.2		1452	49.7	3324	61.9		144	39.7	4928	57.6	
	Houses for rent	317	32.9	1860	26.1		864	29.6	1367	25.4		119	32.8	2264	26.5	
	Public housing	221	22.9	747	10.5		488	16.7	512	9.5		79	21.8	1037	12.1	
	Public housing for disaster	17	1.8	71	1.0		41	1.4	59	1.1		12	3.3	104	1.2	
	Other	26	2.7	156	2.2		77	2.6	112	2.1		9	2.5	216	2.5	
Change of residential school district						<0.001					<0.001					<0.001
	None	454	50.4	4528	65.8		1617	58.4	3504	67.5		189	54.9	5273	64.4	
	Yes	446	49.6	2350	34.2		1152	41.6	1688	32.5		155	45.1	2910	35.6	
Loneliness						<0.001					<0.001					<0.001
	None	317	33.6	6050	85.2		1686	57.8	4862	90.3		171	47.5	6742	80.1	
	Yes	627	66.4	1054	14.8		1229	42.2	521	9.7		189	52.5	1678	19.9	
Community participation						<0.001					<0.001					0.842
	No information of such events	172	18.2	857	12.1		440	15.1	605	11.3		46	13.0	1065	12.6	
	None	665	70.4	4566	64.5		1985	68.2	3383	63.1		233	65.6	5444	64.6	
	Yes	107	11.3	1660	23.4		485	16.7	1377	25.7		76	21.4	1915	22.7	
Decrease in activity opportunities due to COVID-19						<0.001					<0.001					<0.001
	None	355	38.0	3675	52.0		1141	39.4	3027	56.5		116	32.3	4302	51.4	
	Yes	579	62.0	3398	48.0		1754	60.6	2327	43.5		243	67.7	4073	48.6	
Decrease in income due to COVID-19						<0.001					<0.001					<0.001
	None	507	56.3	4602	66.3		1662	59.3	3564	68.0		174	51.0	5359	65.8	
	Yes	394	43.7	2335	33.7		1143	40.7	1677	32.0		167	49.0	2789	34.2	

The Chi-square test of independence (χ2 test) was performed. “None” for loneliness includes never or hardly ever, Yes for sometimes or always. “None” for decrease in activity opportunities due to COVID-19 includes increase or no change. Decrease in income due to COVID-19 includes both large and small decreases.

**Table 4:** Association between the presence of mental health problems and each independent variable.

							*n* = 8966
		Psychological Distress		Insomnia		Probable PTSD	
		OR	95%CI	OR	95%CI	OR	95%CI
Gender (ref: male)							
		1.33	1.13–1.57	1.21	1.08–1.34	1.81	1.41–2.32
The elderly (ref: none)							
		0.90	0.76–1.06	1.36	1.22–1.52	1.49	1.16–1.90
Cohabitant (ref: yes)							
		1.07	0.88–1.31	1.20	1.04–1.38	1.03	0.78–1.37
Temporary housing category (ref: public housing)							
	Prefabricated temporary housing	0.89	0.57–1.39	1.00	0.73–1.37	1.38	0.77–2.48
	Rental	0.78	0.57–1.05	0.90	0.72–1.14	0.86	0.56–1.34
Current residence (ref: owned house)							
	Houses for rent	1.50	1.21–1.87	1.15	0.99–1.33	1.95	1.41–2.69
	Public housing	2.14	1.63–2.83	1.54	1.26–1.88	2.41	1.62–3.58
	Public housing for disaster	2.16	1.08–4.32	1.19	0.73–1.93	2.75	1.23–6.16
	Hospitals and institutions	0.68	0.19–2.43	0.63	0.29–1.36	1.48	0.33–6.53
	Other	1.37	0.76–2.48	1.52	1.04–2.24	1.50	0.64–3.54
Change of residential school district (ref: unknown)							
	None	0.99	0.66–1.50	0.89	0.66–1.20	2.07	1.01–4.28
	Yes	0.93	0.63–1.37	0.89	0.66–1.19	1.55	0.77–3.14
Loneliness (ref: none)		9.08	7.71–10.70	5.55	4.90–6.30	3.52	2.77–4.49
Community participation (ref: yes)							
	No information of such events	1.84	1.36–2.50	1.56	1.29–1.89	0.79	0.52–1.20
	None	1.74	1.36–2.22	1.53	1.34–1.75	0.88	0.65–1.18
Decrease in activity opportunities due to COVID-19 (ref: none)							
		1.41	1.19–1.66	1.86	1.68–2.07	1.80	1.40–2.31
Decrease in income due to COVID-19 (ref: none)							
		1.33	1.12–1.57	1.43	1.28–1.59	1.92	1.51–2.43

Performed binary logistic regression analysis. Variable selection was forced entry. *p* ≥ 0.05 for Hosmer-Lemeshow test results for all logistic regression analyses. The dependent variables were set at 1 (yes, psychological distress; insomnia; probable PTSD) and 0 (no, psychological distress; insomnia; probable PTSD). The independent variables were “gender (male/female)”, “elderly (no/yes)”, “category of temporary accomodation (prefabricated/rental/public)”, “residence (owned/rental/public/public for disaster/hospitals and institutions/other)”, “change of residential school district (no/yes/don’t know)”, “loneliness (no/yes)”, “cohabitants (no/yes)”, community participation (no/yes/no information)”, Decrease in activity opportunities due to COVID-19 (no/yes)”, “Decrease in income due to COVID-19 (no/yes)”. OR: Odds Ratio, 95% CI: 95% Confidence Interval.

## Data Availability

The data presented in this study are available upon request from the corresponding author.
